# Slowdown of synonymous substitution rate preceding the emergence of multiple SARS-CoV-2 variants

**DOI:** 10.1093/ve/veag054

**Published:** 2026-08-20

**Authors:** Jennifer L Havens, Karthik Gangavarapu, Jade C Wang, Faten Taki, Elizabeth Luoma, Jonathan E Pekar, Helly Amin, Steve Di Lonardo, Enoma Omoregie, Scott Hughes, Kristian G Andersen, Tetyana I Vasylyeva, Marc A Suchard, Joel O Wertheim

**Affiliations:** Bioinformatics and Systems Biology Graduate Program, University of California San Diego, 9500 Gilman Dr., La Jolla, CA 92093-0419, United States; Department of Human Genetics, David Geffen School of Medicine, University of California, Los Angeles, 885 Tiverton DriveLos Angeles, CA 90095, United States; Department of Translational Medicine, The Scripps Research Institute, 10550 North Torrey Pines Rd., La Jolla, CA 92037, United States; Bioinformatics and Systems Biology Graduate Program, University of California San Diego, 9500 Gilman Dr., La Jolla, CA 92093-0419, United States; New York City Department of Health and Mental Hygiene, New York City Public Health Laboratory, 455 First Ave, New York 10016, United States; New York City Department of Health and Mental Hygiene, New York City Public Health Laboratory, 455 First Ave, New York 10016, United States; New York City Department of Health and Mental Hygiene, Bureau of Communicable Disease, 42-09 28th St, Long Island City, NY 11101, United States; Bioinformatics and Systems Biology Graduate Program, University of California San Diego, 9500 Gilman Dr., La Jolla, CA 92093-0419, United States; Institute of Ecology and Evolution, University of Edinburgh, Ashworth Building, Edinburgh EH9 3FL,, United Kingdom; New York City Department of Health and Mental Hygiene, New York City Public Health Laboratory, 455 First Ave, New York 10016, United States; New York City Department of Health and Mental Hygiene, New York City Public Health Laboratory, 455 First Ave, New York 10016, United States; New York City Department of Health and Mental Hygiene, New York City Public Health Laboratory, 455 First Ave, New York 10016, United States; New York City Department of Health and Mental Hygiene, New York City Public Health Laboratory, 455 First Ave, New York 10016, United States; New York City Department of Health and Mental Hygiene, Bureau of Public Health Clinics, 455 First Ave, New York 10016, United States; Department of Translational Medicine, The Scripps Research Institute, 10550 North Torrey Pines Rd., La Jolla, CA 92037, United States; Department of Population Health and Disease Prevention, University of California Irvine, 856 Health Sciences Quad, Suite 5600 Irvine, CA 92697-3957, United States; Department of Medicine, University of California San Diego, 9500 Gilman Dr., La Jolla, CA 92093, United States; Department of Human Genetics, David Geffen School of Medicine, University of California, Los Angeles, 885 Tiverton DriveLos Angeles, CA 90095, United States; Department of Biostatistics, Fielding School of Public Health, University of California, Los Angeles, 650 Charles E. Young Dr. South, Los Angeles CA 90095-1772, United States; Department of Biomathematics, David Geffen School of Medicine, University of California, Los Angeles, 5303 Life Sciences, Los Angeles, CA 90095-1766, United States; Department of Medicine, University of California San Diego, 9500 Gilman Dr., La Jolla, CA 92093, United States

**Keywords:** molecular clock, synonymous substitutions, variants, selection, SARS-CoV-2, persistent infections

## Abstract

The emergence of variants has shaped the COVID-19 pandemic. The lack of directly observed precursors to these variants has led to proposals that variants emerge from either persistent infections, transmission in non-human animal populations after reverse-zoonosis, or cryptic transmission in the human population. We investigated the origin of variants by analysing the molecular clock and rate of nonsynonymous and synonymous substitutions in SARS-CoV-2 circulating in human population, persistently infected individuals, non-human animals, and along variant stems: the branches preceding emergence of SARS-CoV-2 variants (Alpha, Beta, Gamma, Delta, Epsilon, Iota, B.1.637, Mu, and Omicron: BA.1, BA.2/BA.4/BA.5). Along the variant stems we find evidence for an acceleration in the non-synonymous substitution rate, as compared with the non-synonymous substitution rate along branches that represent the genetic diversity of circulating virus. We also find evidence for a slowdown in the synonymous substitution rate preceding the emergence of multiple named variants (e.g*.* Beta, Delta, Iota, Mu, Omicron BA.1); a similar pattern was observed in some individuals with persistent infections. However, the synonymous rate slowdown was not observed for all variants, with some exhibiting an increase in synonymous substitution rates preceding their emergence compared with typical viral transmission (e.g*.* Alpha, Epsilon). The similarity in evolutionary dynamics preceding variant emergence and during persistent infections, but not with human to human transmission or during reverse zoonosis, supports the hypothesis that persistent infections were the likely source of many COVID-19 variants.

## 1. Introduction

SARS-CoV-2 emerged in late-2019 and quickly spread across the world. In the ensuing years, lineages with increased propensity for transmission and/or immune escape emerged and were classified as variants of concern and variants of interest. The first named variant, Alpha (B.1.1.7), descended from an early circulating lineage, which we term B.1-like virus. Alpha was characterized by high transmissibility relative to B.1-like virus, largely attributed to N501Y mutation in the spike protein. (Washington et al. 2021, Hill et al. 2022). Starting in late-2020 additional variants with greater transmissibility or immune evasiveness—relative to B.1-like virus—arose, including Beta (B.1.351), Gamma (P.1), Epsilon (B.1.427/B.1.429), Iota (B.1.526), B.1.637, and Mu (B.1.621) (Deng et al. 2021, Faria et al. 2021, Laiton-Donato et al. 2021, Tegally et al. 2021, West et al. 2021). In late 2020, Delta (B.1.617.2) rose to prominence and by mid-2021 became the predominant lineage globally. Shortly thereafter, Omicron (including lineages BA.1, BA.2, BA.4, and BA.5) led to a wave of cases and global weekly infections rose 71% in the last week of December 2021 (Elliott et al. 2021, Lucas et al. 2021, McCrone et al. 2022, Tan et al. 2022, Taylor 2022, Tegally et al. 2022). The succession of new variants influenced SARS-CoV-2 epidemiology and transmission dynamics, increasing the spread of SARS-CoV-2, as well as the selection of public health response strategies. However, the processes leading to variant emergence were never directly observed. Therefore, understanding the evolutionary dynamics preceding variant emergence is of great public health importance, as it can inform surveillance and prevention efforts.

The Alpha, Beta, Gamma, and Mu variant clades are characterized by a long branch preceding their appearance in the phylogenetic tree, representing substantial divergence relative to previously circulating viruses. Similarly, Epsilon, Delta, Iota, and B.1.637 clades are each characterized by a set of branches, without observed intermediates, that form the stem. The variant ‘stem branches’ tend to have more nonsynonymous substitutions than expected given the rate of evolution associated with B.1-like and within-variant clade virus evolution (Deng et al. 2021, Elliott et al. 2021, Faria et al. 2021, Laiton-Donato et al. 2021, Lucas et al. 2021, Washington et al. 2021, West et al. 2021, Hill et al. 2022, McCrone et al. 2022, Tan et al. 2022, Tegally et al. 2022). Although the positive selection and nonsynonymous substitutions on the stem branches of variants have been repeatedly noted, as well as an increase in the overall molecular clock of variant stem branches (Tay et al. 2022), there has only been limited exploration of the behaviour of synonymous and non-coding substitutions (Neher 2022).

SARS-CoV-2 is generally an acute infection, reaching peak viral load within 4 days, and viral clearance typically within 10 days (Jones et al. 2021, Kissler et al. 2023). However, persistent infections lasting longer than 30 days, sometimes lasting longer than a year, have been documented. In a community surveillance study, an estimated 0.7%–3.5% of infections became persistent for longer than 30 days (Ghafari et al. 2024), detected persistent infections are often associated with individuals who are immunosuppressed (Corey et al. 2021, Gonzalez-Reiche et al. 2023, Scherer et al. 2022, Machkovech et al. 2024). Some persistent infections are characterized by accelerated evolution resulting in many nonsynonymous mutations that allowed the virus to evade the host immune response (Machkovech et al. 2024, Ghafari et al. 2025). The stem branches of variants have the same pattern of accelerated nonsynonymous substitutions, and so persistent infections have been proposed as a possible source of variants. It has also been proposed that variants emerge from transmission in non-human animal populations after reverse-zoonosis and cryptic transmission in the human population (Kupferschmidt, n.d.; Wei et al. 2021, Shrestha et al. 2022, Tegally et al. 2022, Markov et al. 2023, Sparrer et al. 2023). Evolution in non-human animals has been suggested because the virus can undergo rapid evolution, which could be unobserved in these undersampled non-human animals (Wei et al. 2021, Sparrer et al. 2023). Cryptic transmission in human populations with poor genomic surveillance could result in long branches as evolution occurs unobserved due to biased undersampling (Shrestha et al. 2022, Tegally et al. 2022).

In this study, we compare the evolutionary dynamics in variant stem branches, persistent infections, and non-human animal hosts with evolution during typical transmission between humans. We consider the stem branches of the Alpha, Beta, Gamma, Delta, Epsilon, Iota, B.1.637, and Omicron variants. Using a Bayesian phylodynamic framework with local molecular clocks, we demonstrate how changes in the rate of synonymous substitutions point to the mechanism behind variant emergence.

## 2. Methods

### 2.1. Data availability

Consensus genomes of publicly available data and analysis used is available in XMLs (Supp Data S1–S23). Consensus genomes or sequencing data is available for query for all samples through NCBI, GISAID, SRA, and COG-UK Consortium. IDs of samples used for B.1-like and variant characterization are available in Supp Table S1. IDs and matching information for persistent infection sequences are available in Supp Table S2. IDs for non-human host sequences are available in Supp Table S3.

### 2.2. SARS-CoV-2 genome surveillance

We analysed SARS-CoV-2 full genome sequences (<1% Ns) from New York City Public Health Laboratory (PHL) collected from 7 February 2020 through 1 June 2022 (*n* = 10 572), previously deposited in GenBank and SRA. We used data from PHL alone because the consensus genome sequences are high quality, the metadata has high fidelity, information on repeated tests is available, and all variant stem branches can be sufficiently characterized even using this subset of global data. Pangolin lineages (v4.1.2) were assigned using UShER (O’Toole et al. 2021). Genomes were aligned with MAFFT (v7.486) (Katoh et al. 2005) to the reference genome Wuhan Hu-1, and coding regions were extracted for further analyses.

We used a random subset of 150 genomes from all genomes not assigned to a Pangolin variant to represent the early background context (B.1-like) (accession numbers are reported in Supp Table S3). We selected smaller datasets to accommodate the Bayesian Robust Count models (see below). Using BEAST (v1.10.5) (Suchard et al. 2018) we inferred a molecular clock rate for these early sequences using only the coding regions of 5.4 x 10^−4^ substitutions/site/year (95% HPD: 4.9 x 10^−4^—5.9 x 10^−4^ substitutions/site/year). We used this rate as a prior for the rate of typical human-to-human transmission in subsequent analyses. We note that the molecular clock rate during typical human to human transmission has slowed over time (Neher 2022). Using this prior across all variant contexts may bias our results as background branches between persistent infections may be underestimated, leading to overestimating the number of person-years experienced by the virus during persistent infections. The slowdown in the human-to-human molecular clock is driven by a slowdown in the rate of nonsynonymous substitutions; the rate of synonymous substitutions has broadly remained unchanged (Neher 2022).

We characterized human-to-human transmission dynamics using surveillance sampling because most evolution is associated with evolution during transmission between acute infections. However, we have not censored from our dataset any evolution that could be associated with onward transmission from a persistent infection, or other distinct mechanisms, as long as it was not considered a variant stem branch. The typical human-to-human transmission we characterize likely includes some evolution from persistent infections that were unidentified, as well as human-to-human transmission of acute infections, so our comparisons between human-to-human transmission and persistent infections are conservative in nature due to the diluted inference.

### 2.3. Characterizing SARS-CoV-2 variant stem branches

**Figure 1:**
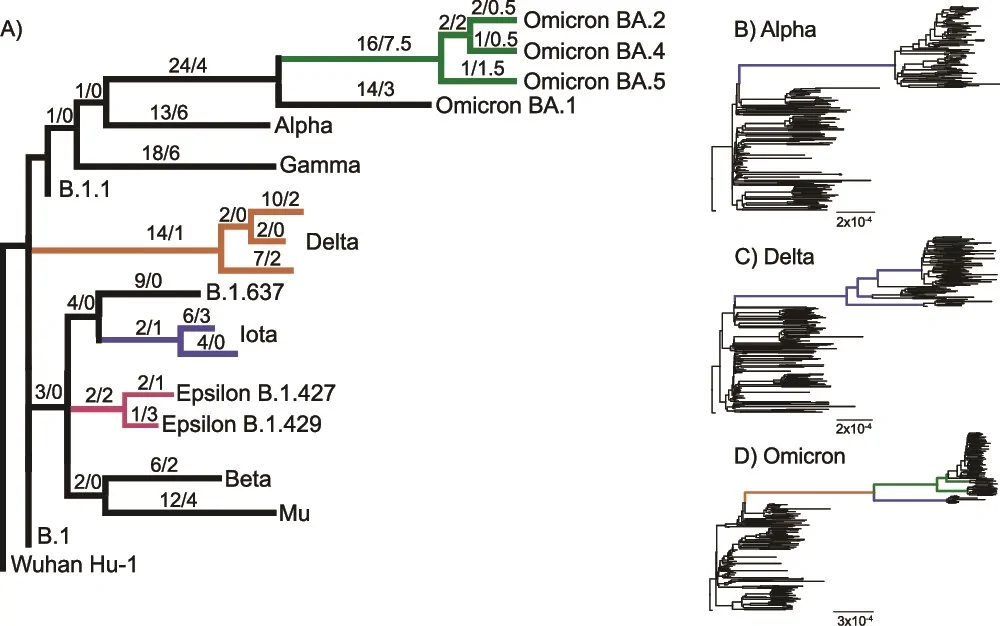
Phylogenetic trees of variant stem branches. (A) Cartoon tree showing SARS-CoV-2 backbone and the relationship between variants, annotated with substitutions formatted as the number of nonsynonymous/synonymous substitutions inferred by robust counting in BEAST. (B) Phylogenetic tree of B.1-like and Alpha sequences, the variant stem branch is highlighted in blue. (C) Phylogenetic tree of B.1-like and Delta sequences, the variant stem branches is highlighted in blue. (D) Phylogenetic tree of B.1-like and Omicron sequences, BA.1 stem branch is highlighted in blue, the stem branches leading to BA.2, BA.4, and BA.5 (BA.2/BA.4/BA.5) is highlighted in green, the base of Omicron, which included in analysis the branches for BA.1 and BA.2/BA.4/BA.5, is highlighted in brown.

For each variant, we used the B.1-like background sequences and all variant sequences to infer a tree with IQTREE (v1.6.12) (accession numbers reported in Supp Table S3) (Minh et al. 2020). Using these trees we defined a variant stem as the path between B.1 and B.1.1 to the base of the variant polytomy expansion (Fig. 1A). For Alpha, Beta, B.1.637, Gamma, and Mu the variant stem was a single branch (Fig. 1B, Supp Fig. S1). The Epsilon variant stem is the branch leading to B.1.427, the branch leading to B.1.429 and their parent branch (Supp Fig. S1E). Delta and Iota variant stem branches are the set of branches preceding the polytomy expansion that is characteristic of variants (Fig. 1A and D, Supp Fig. S1D and F). We subset the Omicron stem branches into two sets for defining the variant stems (BA.1 alone and BA.2, BA.4, and BA.5 combined), but included all Omicron sequences in one alignment for inference. The stem branch of BA.1 is the branch leading to the most recent common ancestor (MRCA) of BA.1, BA.2, BA.4, and BA.5 and the branch leading to the MRCA of BA.1 (Fig. 1D, Supp Fig. S1I). The stem of BA.2, BA.4 and BA.5 is multiple branches leading from the MRCA with BA.1 to the MRCA of each lineage (BA.2, BA.4, BA.5). We chose to combine BA.2, BA.4, and BA.5 because they were closely related and some sites showed repeated changes without observed intermediates (S:452). The base stem for Omicron was included in both the BA.1 stem and the BA.2, BA.4, and BA.5 stem because it represented significant divergence that we felt it was important to include, but could not be reasonably attributed to only BA.1 or BA.2, BA.4, and BA.5 alone.

We note that because each variant stem is defined without other variant sequences there is some shared evolutionary history, i.e*.* from the base of the B.1 polytomy to Beta, Epsilon, Iota, Mu, and B.1.637, and from the bases of B.1.1 to Alpha, Gamma, and Omicron. However, based on ancestral reconstruction <20% of substitutions of any variant are shared with another variant stem (Fig. 1A), with exception of the Omicron variants sharing a base (Fig. 1D) and B.1.637 and Iota sharing more. Delta does not have shared evolutionary history with any other stem.

### 2.4. Persistent intra-host infection phylodynamics

To identify persistent infections, we considered two datasets. First we used public health surveillance data from New York City (NYC), which is contemporaneous with our variant stem data and follows the same sequencing protocols but has little follow up with individuals, and considered cases with genomes sampled more than 30 days apart. Secondly we used data from a longitudinal community surveillance study which had repeated testing of individuals over time, which allows us to focus on longer persistent infections and consider only cases with genomes sampled more than 60 days apart.

For the NYC data we used all genomes from individuals with more than 2 SARS-CoV-2 genomes sampled more than 30 days apart by New York City Public Health Laboratory (PHL), NYC Pandemic Response Laboratory, or Department of Health Wadsworth Center (*n* = 373), this is the repeat genome data set. With the repeat genome dataset and all contemporaneous genomes from PHL (*n* = 10 623) we inferred a phylogenetic tree with IQTREE using the GTR + F + G_4_ substitution model, retaining polytomies, enforcing a minimum branch length of 10^−9^ substitutions/site, and using the fast tree option. We identified persistent infections as individuals that had multiple genomes that formed a cluster (samples could be connected without passing through a node representing a sample from another individual). By using genomes from NYC surveillance, we ensured that persistent infections that are contemporaneous to the variant stems of interest had consistent location and sequencing approach between them; additionally this approach mitigates overrepresentation from immune compromised individuals often seen in convenience sampling. We found a total of 15 individuals with persistent infections, characterized by 42 sequences (accession numbers are reported in Supp Table S4). We then inferred a phylogenetic tree with only the repeat genome data set using the same model as before, except with allnni instead of the fast option, to confirm monophyly; this data was used as the basis for the subsequent Bayesian analysis.

For the UK data we characterized evolution in previously identified persistent infections from the UK (Ghafari et al. 2025). We considered all identified persistent infections with genomes sampled more than 60 days apart (23 individuals and 62 sequences) and available from archived COG consortium sequences, accessed 2026-02-03 (accession numbers are reported in Supp Table S4). We chose a longer threshold for persistent infection in this data set as compared with the NYC data, because longer persistent infections allow for more signals to accumulate which is beneficial to a robust analysis and there are enough cases that still meet this stricter criteria. Monophyly of persistent infections was confirmed using UShER to place the persistent infection genomes on the global SARS-CoV-2 tree (Turakhia et al. 2021) accessed through the UCSC browser 2026-02-03. NYC genomes of the same variant were used as background context.

For each case we inferred a local clock (Yoder and Yang 2000) with BEAST, using sequences of the same variant, or the B.1-like subset, as context (Supp Fig. S2).

### 2.5. Deer and mink virus genomes

To investigate SARS-CoV-2 evolution associated with a deer host we considered 5 clades, comprising 22 previously published genomes (McBride et al. 2023). We choose clades with more than 0.33 years of evolution inferred in previous analysis (Supp Fig. S3). To investigate SARS-CoV-2 evolution associated with a mink host we considered 244 viral genomes from one introduction into mink that circulated for more than 33 months (Crespo-Bellido et al. 2026). We included in the background context: NYC Delta background context and the 4 closest human sequences identified by placing mink sequences on the global SARS-CoV-2 tree using USHER. Sequence IDs are available (Supp Table S5).

### 2.6. Bayesian local clock inference and robust counting

We inferred a local molecular clock for each variant stem, persistent infection, and deer clade using BEAST with a GTR for each codon position (Supp Data S1–S23). With the joint time and codon model we used robust counting (O’Brien et al. 2009) to infer the expected number of synonymous and nonsynonymous substitutions on branches over time, consistent with prior approaches of joint inference (Lemey et al. 2012). For each variant stem we used the B.1-like background sequences for context together with all variant sequences. For persistent infections and deer clades, all sequences of the same variant from PHL were used for context. Context sequences (variant and B.1-like) were given a tight molecular clock prior (5.4 x 10^−4^ substitutions/site/year, standard deviation: 2.3 x 10^−5^), inferred from the B.1-like context. We inferred a local clock on the stem of the variant clades, and within the persistent infection and deer clades. For Omicron, the base branch (parent branch of BA.1, BA.2, BA.4, and BA.5) shared a local clock with the BA.1 stem, while the stem of BA.2, BA.4, and BA.5 was an independent local clock. We included the base branch for both stems in the post-BEAST analysis. The local clock was set as the product of the background rate and an increment factor with a log normal prior centered on 1. For each analysis we ran 2 chains for 5 x 10^8^ to 10^9^ states and investigated the chain convergence using Tracer (v1.7.2) (Rambaut et al. 2018), then used LogCombiner (v10.5.0) (Suchard et al. 2018) to resample and combine the obtained tree distributions at a lower frequency. We did not consider insertions, deletions, or noncoding regions.

For each set of branches of interest we summarized from the joint posterior the person years (i.e. sum of the length of branches of interest), clock rate, synonymous substitution rate, and nonsynonymous substitution rate as compared to the background context. The posterior probability that the rate is larger or smaller than the background rate is calculated by counting the number of states in which the local rate is greater than the background for each tree in the posterior, and taking the maximum of the proportion of states that are greater, or one minus that proportion (e.g. the minimum posterior is 0.5). In our interpretation, we considered only variant stems, persistent infections from NYC, and deer clades with more than 0.25 person years, and only persistent infections from the UK with more than 0.66 person years. The variant under monitoring B.1.1.519 and 22 (5 from NYC and 17 from UK) persistent infections were excluded due to this criteria.

### 2.7. Inference of ω and positive selection

Using BUSTED (v2.5.42) (Murrell et al. 2015), we inferred the ω or dN/dS, and tested for positive selection on the branches of interest described above on the same phylogenetic trees. All other branches were considered part of a nuisance set.

## 3. Results

To contextualize the origin of SARS-CoV-2 variants, we assembled genomic datasets representing four modes of SARS-CoV-2 evolution: typical human-to-human transmission (i.e. background molecular clock rate), long-term persistence within individuals, transmission between non-human animals following reverse zoonosis, and the phylogenetic stem branches preceding emergence of variants.

### 3.1. SARS-CoV-2 variant stem branches

We identified the stem branches preceding the B.1 variants (Beta, Delta, Epsilon, Mu, Iota, and B.1.637) and B.1.1 variants (Alpha, Gamma, Omicron) on a maximum likelihood phylogenetic tree with 10 572 high quality SARS-CoV-2 genomes representing circulating genomic diversity in New York City (NYC), over a 2 year period (Fig. 1). We focused on NYC because it is a major urban center with robust public health surveillance that produced a large volume of high-quality surveillance of SARS-CoV-2 genomes reflecting global viral diversity, including all variants of interest. Branches that were not associated with a variant stem were considered to represent evolution associated with typical human-to-human transmission, including both within variant clades and all branches that preceded the evolution of variants (such as B.1 and B.1.1 virus), which we term B.1-like virus. For each variant, we compared the evolution on the stem branches to evolution within B.1-like phylogeny and the variant clade. For each variant (Fig. 1, Supp Fig. S1), we applied local molecular clocks on variant stem branches in a Bayesian phylodynamic inference framework.

### 3.2. Identification of persistent SARS-CoV-2 infections

**Figure 2:**
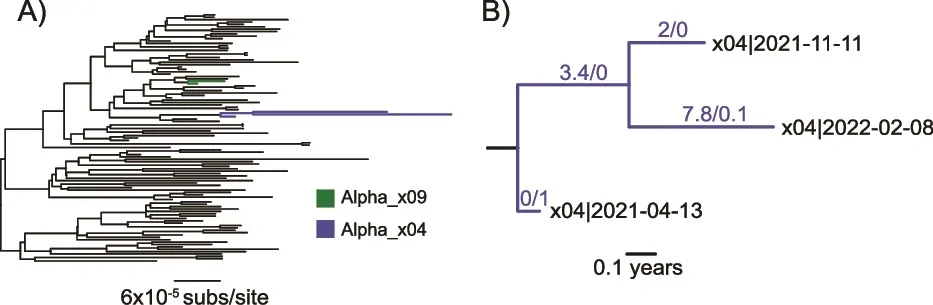
Phylogenetic trees of persistent infections. (A) Phylogenetic tree of Alpha sequences, persistent infection clades are highlighted in blue (Alpha_x04) and green. (B) Phylogenetic tree of persistent infection Alpha_x04 with time scaled branches annotated with robust counts of nonsynonymous/synonymous substitutions.

To identify persistent SARS-CoV-2 infections, we inferred a phylogenetic tree from viral genomes sampled from individuals in New York City (*n* = 10 623) including those with two or more genomes sampled > 30 days apart (genomes *n* = 396, individuals *n* = 194). We defined persistent infections as instances in which viral genomes sampled from the same individual were monophyletic. This approach yielded persistent infections that were contemporaneous to the stem branches of the named variants with consistent location and sequencing approach between all the genomes. We found 15 individuals with persistent infections, represented by 46 viral genomes. The remaining 181 individuals were determined to most likely be instances of re-infection. Seven people with persistent infections had viral evolution comprising more than 3 person months of evolution across the phylogeny; thus we only included these seven individuals in the subsequent analyses. These viral genomes were B.1-like (*n* = 2), Alpha (*n* = 1), Delta (*n* = 2), Iota (*n* = 1), and B.1.637 (*n* = 1). Additionally, we analysed 23 persistent infections from the United Kingdom (UK) that were previously identified (Ghafari et al. 2025). Of these, 7 had more than 8 person months of evolution and were included in subsequent analyses. We applied a local molecular clock for viruses from each individual with a persistent infection in Bayesian phylodynamic inference (Fig. 2; Supp Fig. S2) and compared the evolution during these persistent infections with evolution within their respective variants.

### 3.3. SARS-CoV-2 clades in non-human hosts

To characterize SARS-CoV-2 evolution in non-human animals after reverse zoonosis, we used 22 viral genomes from clades of deer SARS-CoV-2 arising from 5 previously characterized reverse zoonosis events (McBride et al. 2023) and 244 viral genomes from one introduction into mink (Crespo-Bellido et al. 2026). All deer and mink virus samples were descended from Alpha and Delta variants. We applied a local molecular clock for each non-human host clade in Bayesian phylodynamic inference in the phylogenetic context related to human virus sequences from their respective Alpha and Delta variants (Supp Fig. S3).

### 3.4. SARS-CoV-2 synonymous substitution rate

To understand the evolutionary dynamics in different contexts we inferred local molecular clocks of coding regions in a phylodynamic framework (Suchard et al. 2018). We used robust counting to infer the synonymous substitutions along the tree and infer the rate of synonymous substitution (i.e. synonymous substitutions across all coding sites/year) stratified by partitions of tree branches (O’Brien et al. 2009, Lemey et al. 2012). Along each foreground partition (i.e. variant stem branches, persistent infection clades, and deer clades) and the background partition (associated with typical human-to-human transmission), the rate of substitutions was calculated as the sum of inferred synonymous substitutions divided by the cumulative branch length. The inferred background rate of synonymous substitutions along internal branches in coding regions of B.1-like virus was 6.8 substitutions/year [95% highest posterior density (HPD): 6.1–8.2 substitutions/year] (Supp Table S4).

### 3.5. Synonymous substitution rate preceding variants

**Figure 3:**
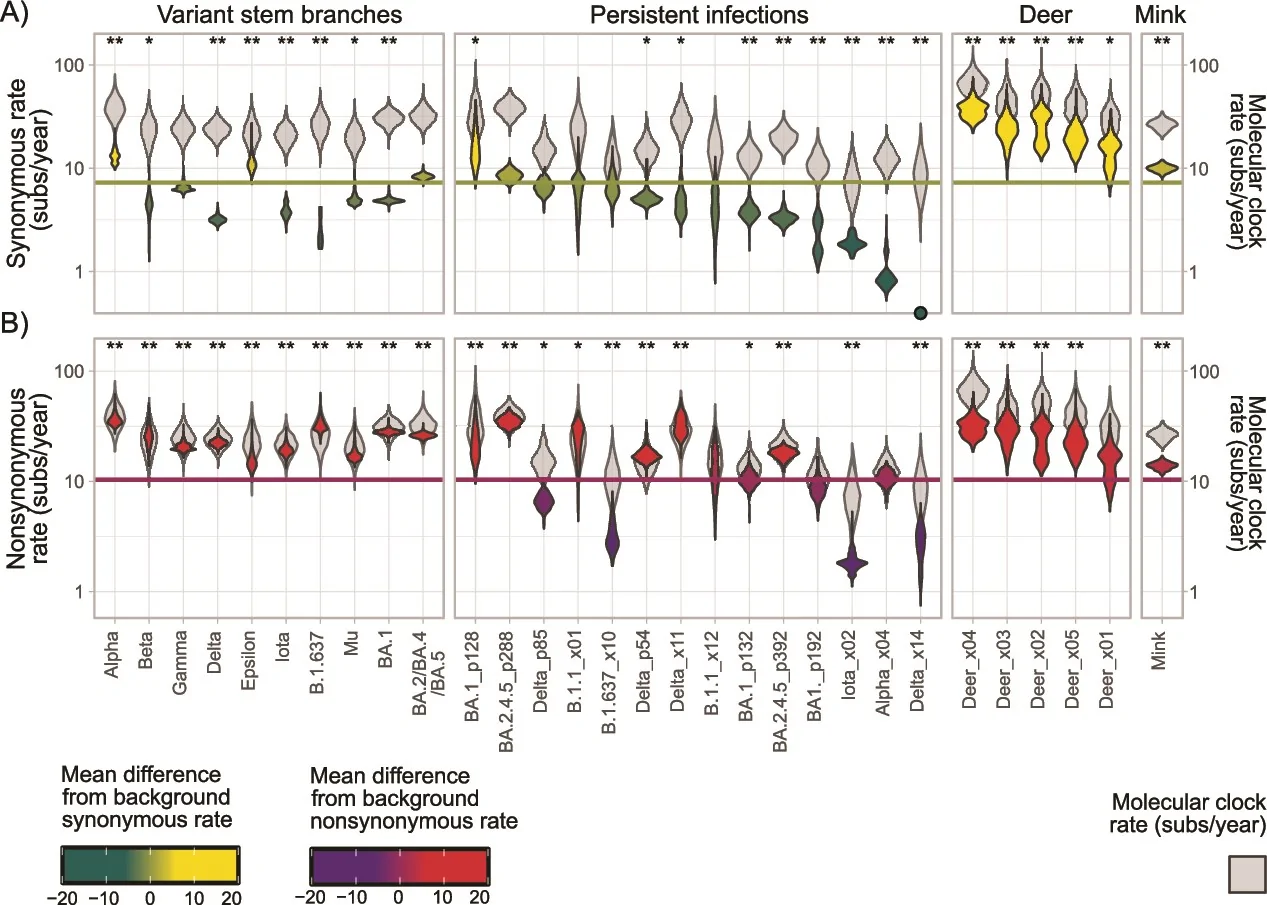
Rate of nonsynonymous and synonymous substitutions and the molecular clock. (A) Summary of foreground synonymous substitution rate (coloured) and overall molecular clock rate (grey) with posterior probability that the rate is larger or smaller than the background synonymous rate (^*^P > .9; ^**^P > .99). Synonymous substitution rate (foreground) shaded by the difference of mean synonymous substitution rate of foreground and mean synonymous rate of background. Background median synonymous substitution rate (6.8 substitutions/year) denoted by a solid horizontal line. Note that Delta_x14 had 0 synonymous substitutions/year and is plotted below the log scale. (B) Summary of foreground nonsynonymous substitution rate (coloured) and overall molecular clock substitutions rate (grey) with posterior probability that the nonsynonymous rate is larger or smaller than the background nonsynonymous rate (^*^P > .9; ^**^P > .99). Nonsynonymous substitution rate (foreground) shaded by the difference of mean nonsynonymous substitution rate of foreground and mean nonsynonymous rate of background. Background median nonsynonymous substitution rate (10.1 substitutions/year) denoted by solid horizontal line.

We then compared the synonymous substitutions rate on the stem branches preceding variants with the synonymous substitutions rate during typical B.1 transmission and within the corresponding variant clade. We observed a pattern along the stem branches leading to Beta, Delta, Iota, B.1.637, Mu, and BA.1, whereby the rate of synonymous substitutions was significantly slower than the inferred background synonymous rate (Fig. 3A). For example, the stem branches of the Delta variant encompass 1.6 person years, with an expectation of ~11 synonymous substitutions (95% HPD: 9.5–12.8); we only observed 5 synonymous substitutions (Supp Table S5), less than half of the amount expected. The synonymous substitution rate was 3.2 substitutions/year (95% HPD: 2.8–3.8 substitutions/year). Similarly for the Iota variant, we observed only 4 synonymous substitutions (Supp Table S2) over the 1.1 person-years comprising the variant stem branches; in contrast, under the synonymous substitutions rate during human-to-human transmission, we would have expected 7 synonymous substitutions (95% HPD: 6.0–9.2 substitutions) over this time period. We observed a synonymous substitution rate of 3.8 substitutions/year (95% HPD: 2.9–5.1 substitutions/year) along the Iota stem branches. Similarly in the stem of BA.1 the expected number of synonymous substitutions is 10.2 (95% HPD: 9.2–11.2 substitutions), however only 7 synonymous substitutions are observed on the BA.1 stem branches.

In contrast to most variants we examined, there is evidence of increased rate of synonymous substitutions in the Alpha and Epsilon stem branches (Fig. 3A). For example, the Alpha stem branch represents 0.43 person years of evolution and has 6 synonymous substitutions, double the expected 3.0 synonymous substitutions (95% HPD: 2.3–3.5 substitutions). The Alpha stem branch had a rate of 13.1 synonymous substitutions/year (95% HPD: 10.0–17.7 substitutions/year).

We did not detect an increase or decrease in the synonymous substitution rate on the Gamma or BA.2/BA.4/BA.5 stem branches (Fig. 3A). We inferred a rate of 6.4 substitutions/year (95% HPD: 5.6–7.9 substitutions/year) leading to Gamma and 8.3 substitutions/year (95% HPD: 7.3–9.2 substitutions/year) leading to BA.2/BA.4/BA.5, which is consistent with the background rate of 6.8 synonymous substitutions/year (95% HPD: 6.1–8.2 substitutions/year).

### 3.6. Synonymous substitution rate during persistent infections

When we considered persistent infections, 8 of the 14 persistent infections had a slower rate of synonymous substitutions compared with the background (Supp Table S1 and Fig. 3A). For example in the case of Alpha_x04 (Supp Fig. S2B), there was 1 synonymous substitution over 1.2 person years and the synonymous rate was 0.8 substitutions/year (95% HPD 0.6–1.8 substitutions/year). This rate was substantially slower than the synonymous substitution rate of 6.8 synonymous substitutions/year in the context of human-to-human transmission. This slowdown in synonymous substitution rate is comparable to that seen in the Beta, Delta, Iota, B.1.637, Mu, and BA.1 variant stem branches.

In 5 of the 14 persistent infections, the rate of synonymous substitutions was not significantly different from the background (Fig. 3A). For example, B.1.637_x10 had 2 synonymous substitutions over 0.32 person years and a synonymous substitution rate of 6.4 substitutions/year (95% HPD: 3.9–10.5 substitutions/year), which is consistent with the synonymous substitutions rate expected during typical transmission.

One persistent infection, BA.1_p128, had a faster rate of synonymous substitutions compared with the background. There was close to a full year of evolution (0.98 person years) in the case of BA.1_p128 and a rate of 14.6 synonymous substitutions/year (95% HPD: 7.5–29.8 substitutions/year); this rate largely overlaps with the Alpha stem rate inference of 13.1 synonymous substitutions/year (95% HPD: 10.0–17.7 substitutions/year).

### 3.7. Synonymous substitution rate in non-human host virus

We also compared typical transmission to evolution within clades of SARS-CoV-2 sampled in deer and mink. We found that there was an increased rate of synonymous substitutions in all 4 deer clades compared with human-to-human transmission (Fig. 3A and Supp Table S1). For example, deer clade Deer_x05 had 17 synonymous substitutions over 0.91 deer infection years and a rate of 19.1 synonymous substitutions/year (95%HPD: 11.4–30.2), which is faster than expected during transmission within human hosts. SARS-CoV-2 in mink had a similar pattern, with an increase in the synonymous substitutions relative to typical human transmission: 10.0 synonymous substitutions/year (95%HPD: 8.485–11.45) over 6.6 mink years of evolution.

### 3.8. Nonsynonymous substitution rate

We inferred the rate of nonsynonymous substitutions over time (i.e. nonsynonymous substitutions across all coding regions/year) using the same approach used to infer the rate of synonymous substitutions. Using B.1-like virus genomes to represent typical human-to-human transmission we inferred a background rate of 10.1 nonsynonymous substitutions/year (95% HPD: 9.1–11.2 substitutions/year).

All variant stem branches that we considered had an elevated rate of nonsynonymous substitutions relative to the background (Fig. 3B; Supp Table S1). For example, the Alpha stem branch had a rate of 35.5 nonsynonymous substitutions/year (95% HPD: 29.7–45.6 substitutions/year) with 15 nonsynonymous substitutions over 0.43 person years (Fig. 1). The stem branches of Iota which had 22 nonsynonymous substitutions over 1.1 person years with a rate of 19.0 nonsynonymous substitutions/year (95% HPD: 15.3–23.6 nonsynonymous substitutions/year), which exceeds the expected rate of nonsynonymous substitutions. The excess number of nonsynonymous substitutions that has been previously described (West et al. 2021, Hill et al. 2022).

In persistent infections, 7 of 14 had an increased rate of nonsynonymous substitutions compared with the rate associated with human-to-human transmission. One of these persistent infections of increased rate was Delta_x11, which had a rate of 31.2 nonsynonymous substitutions/year (95% HPD: 22.3–46.2 substitutions/year). This increased rate is consistent with the dynamics observed on variant stem branches. We observed high variance between nonsynonymous substitution rates in persistent infections, consistent with previous findings (Ghafari et al. 2025). Among 14 persistent infections, 4 had a slower rate of nonsynonymous substitutions compared with rates under human-to-human transmission. For example, Iota_x02 had 1 nonsynonymous substitution in 0.55 person years and a nonsynonymous substitution rate of 1.8 nonsynonymous substitutions/year (95% HPD: 1.3–2.6 substitutions/year). The remaining 3 persistent infections we considered had a similar rate of nonsynonymous substitutions compared to the background. For example, Alpha_x04 had a rate of 10.7 nonsynonymous substitutions/year (95% HPD: 8.4–13.6 substitutions/year), which is consistent with the rate of nonsynonymous substitutions during typical transmission: 10.1 nonsynonymous substitutions/year.

McBride et al. (2023) found an increased rate of nonsynonymous substitutions in deer; our approach recapitulated this finding (Fig. 3B). For example, the clade Deer_x05 had a rate of 22.4 nonsynonymous substitutions/year (95% HPD: 13.1–35.3 substitutions/year), with 20 nonsynonymous substitutions over 0.91 deer infection years. Virus from mink infections also showed an increased rate of nonsynonymous substitutions.

### 3.9. Molecular clock and selection in human-to-human transmission and preceding variants

We considered the interaction of nonsynonymous and synonymous substitutions by estimating the overall molecular clock, which is the nonsynonymous and synonymous rates combined, and the ratio of these rates when normalized [nonsynonymous substitutions per nonsynonymous site (dN) and synonymous substitutions per synonymous site (dS)],dN/dS or ω. The ω measure is a useful and informative statistic describing the nature of selective forces. We inferred ω and tested for positive selection using BUSTED (Murrell et al. 2015), and inferred overall clock rates from the Bayesian analysis. We inferred the background rate of evolution and ω associated with human-to-human transmission using B.1-like sequences. The overall rate of substitutions in coding regions was 5.5 x 10^−4^ substitutions/site/year (95% HPD: 5.1 x 10^−4^—6.2 x 10^−4^; Supp Table S1), and ω was 0.63.

Variant stem branches tended to have a molecular clock rate that was elevated or comparable to the background clock rate, and stem branch ω was typically greater than the background ω (Table 1; Supp Fig. S4). In the variant stem branches we observed that nonsynonymous substitutions (Fig. 3B red violin) provided a larger contribution to the variation in the overall molecular clock (Fig. 3 grey violin), whereas the synonymous substitutions (Fig. 3A green violin) contributed a lower proportion to the variation in the molecular clock (Fig. 3 grey violin); this observation is consistent with a high dN/dS or ω seen in the variant stem branches. The Alpha, Gamma, and the BA.2/BA.4/BA.5 stem branches had an increase in the overall rate and ω, largely driven by higher nonsynonymous substitutions rates (Fig. 3B; Supp Table S1). The stem branches of Beta, Delta, Iota, B.1.637, Mu, and BA.1 variants also had an increase in nonsynonymous rates; however, due to a decrease in the synonymous substitution rates, the overall molecular clocks did not increase as much, compared with the other variants (Fig. 3; Supp Table S1). The decrease in synonymous rate explains the noted lack of speed up in the overall molecular clock of the Delta stem branches despite the same excess of nonsynonymous substitutions that is seen in the Alpha stem branch (Hill et al. 2022). The increase in ω was driven by both the increase in nonsynonymous and decrease in synonymous substitutions rates (Table 1). Although, we note that 95% HPD of the B.1.637 stem branch included zero synonymous substitutions, which makes inference of ω difficult (Supp Table S2).

**Table 1:** Estimate of ω and evidence for positive directional selection.

Category		Time	ω	*P*
		(person years)		
Background	–		0.63	
Variant stem branches	Alpha	0.43	1.2	< .01^*^
	Beta	0.45	2.34	.1
	Gamma	0.94	1.32	< .01^*^
	Delta	1.58	2.84	.02^*^
	Epsilon	0.54	0.31	.42
	Iota	1.07	3.82	< .01^*^
	B.1.637	0.51	30.22^a^	< .01^*^
	Mu	0.82	1.44	.39
	BA.1	1.45	2.39	.03^*^
	BA.2/BA.4/BA.5	1.91	1.37	< .01^*^
Persistent infections	Alpha_x04	1.24	5.27	.05
	B11_x01	0.32	1.64	.33
	B11_x12	0.49	1.23	.48
	B1637_x10	0.32	0^b^	N/A^c^
	Delta_x14	0.36	40.99^a^	.29
	Delta_x11	0.5	3.04	.12
	Delta_p85	0.92	0.54	N/A^c^
	Delta_p54	0.8	15.64	< .01^*^
	Iota_x02	0.55	31.43^a^	.34
	BA1_p128	0.98	0.57	N/A^c^
	BA1_p132	1.1	1.07	.3
	BA1_p192	0.66	10.48	< .01^*^
	BA.245_p288	0.95	2.32	.06
	BA.245_p392	1.23	2.91	.06
Deer clades	Deer_x04	1.23	0.39	N/A^c^
	Deer_x05	0.91	0.54	.01^*^
	Deer_x01	1.3	0.43	N/A^c^
	Deer_x02	0.82	0.39	N/A^c^
	Deer_x03	0.45	0.48	N/A^c^
Mink clade	Mink	6.6	0.57	.03^*^

^a^95% HPD includes 0 synonymous substitutions.

^b^95% HPD includes 0 nonsynonymous substitutions.

^c^Unconstrained model does not include positive selection.

^*^ *P* < .05, BUSTED test for positive selection.

The Epsilon stem branches did not show the same behaviour as other variants, as it was the only variant stem branches where ω was not higher than the background, synonymous and nonsynonymous substitutions were both large contributors to the overall clock rate, indicated by the overlap in the synonymous substitution rate with the overall molecular clock rate (Fig. 3A). The overall molecular clock rate of the Epsilon stem branches was not significantly different from the background rate, unlike other variant stem branch dynamics.

### 3.10. Molecular clock and selection during persistent infections

In the 14 persistent infections we analysed, 11 had an increase in ω compared with the background (Table 1), and 6 had a change in the molecular overall clock rate. Some persistent infections had changes in rates that are similar to that in variant stem branches. For example BA.2/4/5_p392, over the course of 1.23 person years of evolution, had no change in the overall molecular clock rate, a slowdown in the synonymous substitution rate, an increase in nonsynonymous substitution rate, and increase in ω relative to typical transmission, the overall molecular clock was primarily driven by nonsynonymous substitutions (Fig. 3A and Supp Table S2). This change in rates is consistent with Beta, Iota, and Mu stem branches. Delta_x14 had the same patterns of rate changes as Delta, B.1.637, and BA.1; an increase in the overall rate, increase in ω, decrease in the synonymous substitution rate and increase in the nonsynonymous substitution rate.

While most persistent infections had an increase in ω, no change in overall clock, and a decrease or no change in the synonymous substitution rate relative to the background, there was high variability. Most persistent infections had an increase in ω relative to the background; in contrast Delta_p85 showed a decrease in ω driven by a decrease in the nonsynonymous rate. Persistent infections including Iota_x02 showed a slowdown in the overall molecular clock, with a slowdown in both the synonymous and nonsynonymous rates; despite the decrease in the nonsynonymous rate, ω increased relative to the background. Finally, BA.1_p128 showed the same pattern of an overall increase in rate (synonymous, nonsynonymous, and overall) as the Alpha stem. Overall we found persistent infections can have the same changes in the molecular clock and ω compared with the background that was characteristic of variant stem branches.

### 3.11. Molecular clock and selection in deer and mink virus

In the 4 clades of SARS-CoV-2 sampled from deer and one from mink, we saw a consistent pattern of an increase in nonsynonymous and synonymous substitution rate, and a corresponding increase in the overall rate of substitutions (Table 1; Fig. 3). This increased rate is consistent with previous findings (McBride et al. 2023). The selective pressure across all deer and mink clades, assessed by ω, tended to be similar to the background, because the nonsynonymous and synonymous rates increased relatively proportionally. This finding is consistent with a lack of gene wise positive selection previously described (McBride et al. 2023). This pattern of equal contribution of synonymous and nonsynonymous substitutions to the overall clock rate is not consistent with that observed in the variant stem branches (Fig. 3).

## 4. Discussion

We performed phylodynamic analysis to understand the evolutionary dynamics of the phylogenetic branches ancestral to SARS-CoV-2 variants. We found evidence for a slowdown in the absolute rate of synonymous substitutions preceding the emergence of several variants (Beta, Delta, Iota, and Omicron), accompanied by an increase in the absolute rate of non-synonymous substitutions in all variants. We find that persistent infection can have detectable changes in synonymous and nonsynonymous rates that are consistent with this pattern. In contrast, no such slowdown in the synonymous substitution rate was observed during typical transmission between humans or between deer or mink after reverse-zoonosis. These findings support the conclusion that SARS-CoV-2 variants could arise during persistent infection and do not support either an animal reservoir or during cryptic human-to-human transmission as the origin of variants.

Variant stem branches have different rates of evolution and selection dynamics compared to typical human-to-human transmission, suggesting cryptic transmission in the human population is not the source of SARS-CoV-2 variants. The overall molecular clock of some variant stem branches (e.g. Delta) is consistent with human-to-human transmission, leading to the suggestion that variants like Delta could have emerged from typical transmission (Hill et al. 2022). However, our analysis uncovered that this molecular clock is not consistent with human-to-human transmission when we consider that a slower rate of synonymous substitutions is obfuscating an increase in nonsynonymous substitutions. The rate of synonymous substitutions associated with typical transmission within variant clades has broadly remained unchanged throughout the pandemic (Neher 2022).

Together the molecular clock, selection dynamics, and deer/mink characteristic mutations suggest that reverse zoonosis to deer or mink is not the primary source of variants. Deer and mink are the only non-human hosts which are known to have experienced sustained circulation of SARS-CoV-2 (Holmes 2024). SARS-CoV-2 in both deer and mink following reverse zoonosis exhibited an increased rate of both nonsynonymous and synonymous substitutions, and selection pressure similar to human transmission; these selection dynamics are distinct from the dynamics observed on most variant stem branches. Additionally, SARS-CoV-2, circulating in deer and mink, tends to acquire characteristic substitutions which are not seen in variant stem branches (Hale et al. 2022, McBride et al. 2023). We cannot exclude the possibility that infection in non-human animals could be the source of SARS-CoV-2 variants, particularly given that we only analysed SARS-CoV-2 in deer and mink. However, SARS-CoV-2 evolution in deer and mink does not recapitulate the evolutionary dynamics preceding the emergence of variants.

We observed changes in the nonsynonymous substitutions rate in persistent infection and variant stem branches. The increased rate of nonsynonymous substitutions in variant stem branches has been attributed to selection pressure (Volz et al. 2021, Hill et al. 2022, McCrone et al. 2022). In persistent infections there is greater variance in the nonsynonymous substitution rate than in the synonymous substitutions rate (Harari et al. 2024, Ghafari et al. 2025), although high variability between persistent infections has been observed (Ben Zvi et al. 2025). This larger variance in nonsynonymous substitutions can be explained by the disproportionate impact of selection on nonsynonymous substitutions and higher rate of substitutions. Mutations associated with VOCs have been observed arising in persistent infections (Joseph et al. 2025). Some persistent infections have an increased observed rate of nonsynonymous substitutions, which is consistent with variant stem branches, and unlike human-to-human transmission.

We found some persistent infections appear to have slower rates of synonymous substitutions, as was seen in a subset of variant stem branches. A slow rate of evolution could be explained by lingering non-replicating and non-viable RNA (Ghafari et al. 2024); however, we note during continued evolution there are fewer synonymous substitutions than expected on branches across the full Alpha_x04 clade (Fig. 1B) and BA.2.4.5_p392 (Supp Fig. S5) suggesting continued replication of virus with slower synonymous substitutions. A change in synonymous substitution rate is often associated with a change in viral replication rate (Jenkins et al. 2002, Holmes 2003, Hanada et al. 2004) synonymous substitution rate can also be impacted by factors such as selection, and mutation rate (Pond and Muse 2005).

Observed substitution rates associated with inter-host evolutionary dynamics are driven by bottlenecks at the time of transmission, in which relatively low frequency mutations in the transmitting host can become high frequency in the recipient, associated with founder effects (McCrone et al 2018). In contrast, intrahost variation does not necessarily experience the same bottleneck and mutations that do not have a high impact on fitness, including many synonymous substitutions, are not likely to become majority substitutions. In SARS-CoV-2 subpopulations harbouring mutations can rise and decline in frequency during persistent infection; if these mutations do not become the majority, this diversity will not be observed in the consensus sequence (Ghafari et al. 2025; Velasquez-Reyes et al. 2025). In the context of onward transmission of a persistent infection, only a portion of the diversity that accumulates over time will be passed onto the recipient. In persistent infections the lack of serial bottlenecks could hypothetically cause a slowdown in the rate at which synonymous substitutions accumulate at the consensus level (i.e. a slowdown in the synonymous substitution rate), as mutations not under positive selection fail to become fixed—consistent with our observations; whereas mutations at the sub-consensus level continue to accumulate at a rate consistent with inter-host rates—consistent with previous observations (Ben Zvi et al. 2025, Ghafari et al. 2025).

SARS-CoV-2 primarily infects cells in the upper respiratory tract, but can move to other areas of the body, where it experiences distinct selection patterns (Jary et al. 2020, Rueca et al. 2020, Van Cleemput et al. 2021, El Moussaoui et al. 2024). Synonymous substitution rate and viral replication can be affected by host species (Kim et al. 2022), cell tropism (Hicks and Duffy 2014) and virus sequestration (Keita et al. 2021). In HIV, the synonymous substitution rate varies between infections and impacts the dynamics of the infection (Lemey et al. 2007). The inferred slowdown in the synonymous substitution rate preceding variants could potentially be a consequence of persistent infection in non-respiratory cells. Understanding how SARS-CoV-2 evolves in different areas of the body will require more samples from the diverse body compartments and experimental testing. Viral genomics from wastewater suggests that SARS-CoV-2 experiences distinct patterns of evolution in gut tissue; however, the vast majority of SARS-CoV-2 genomes available for analysis are from the upper respiratory tract (Machkovech et al. 2024).

A clue to the slowdown in synonymous substitution rates may be found in another virus. Ebola virus, although typically causing an acute infection, can persist in immunoprivileged sites where it underwent substantially slower rates of replication (Whitmer et al. 2018). Transmission after long-term persistence has seeded multiple Ebola outbreaks, including those where the stem branch leading to the outbreak had fewer synonymous substitutions than expected given the amount of time of evolution (Keita et al. 2021). SARS-CoV-2 can replicate in different body compartments (Van Cleemput et al. 2021) and, like Ebola virus, produce persistent infections that can exhibit slower rates of synonymous substitutions. These observations, suggests that it is possible that SARS-CoV-2 may have experienced slower replication rates in additional anatomical compartments during persistent infection.

In the stem branches preceding most named SARS-CoV-2 variants, we observed decreased synonymous substitution rates and in all variant stems we observed increased nonsynonymous substitution rates. Synonymous and nonsynonymous substitution rates consistent with the trends inferred on these variant stems were observed in some persistent infections, but not during typical human-to-human transmission or in deer or mink hosts. The novel observation of a slowdown in synonymous substitutions in variant stems provides evidence supporting persistent infections as a source of variants although the mechanism that causes this dynamic is unclear. The likely contribution of persistent infections in variant emergence highlights the importance of further research into the dynamics and prevention of persistent SARS-CoV-2 infections.

## Supplementary Material

Supplementary_material_veag054(1)
