# Supplementary material for: Slowdown of synonymous substitution rate preceding the emergence of multiple SARS-CoV-2 variants
Source: Virus Evol. 2026 Aug 20;12(1):veag054. doi: 10.1093/ve/veag054 (PMC13580085; doi:10.1093/ve/veag054)
Supplement: Supplementary file 1 [file supplementary_material_veag054(1).zip › Supplementary_material_veag054/SuppFig1_StemSuppFigTrees.pdf]

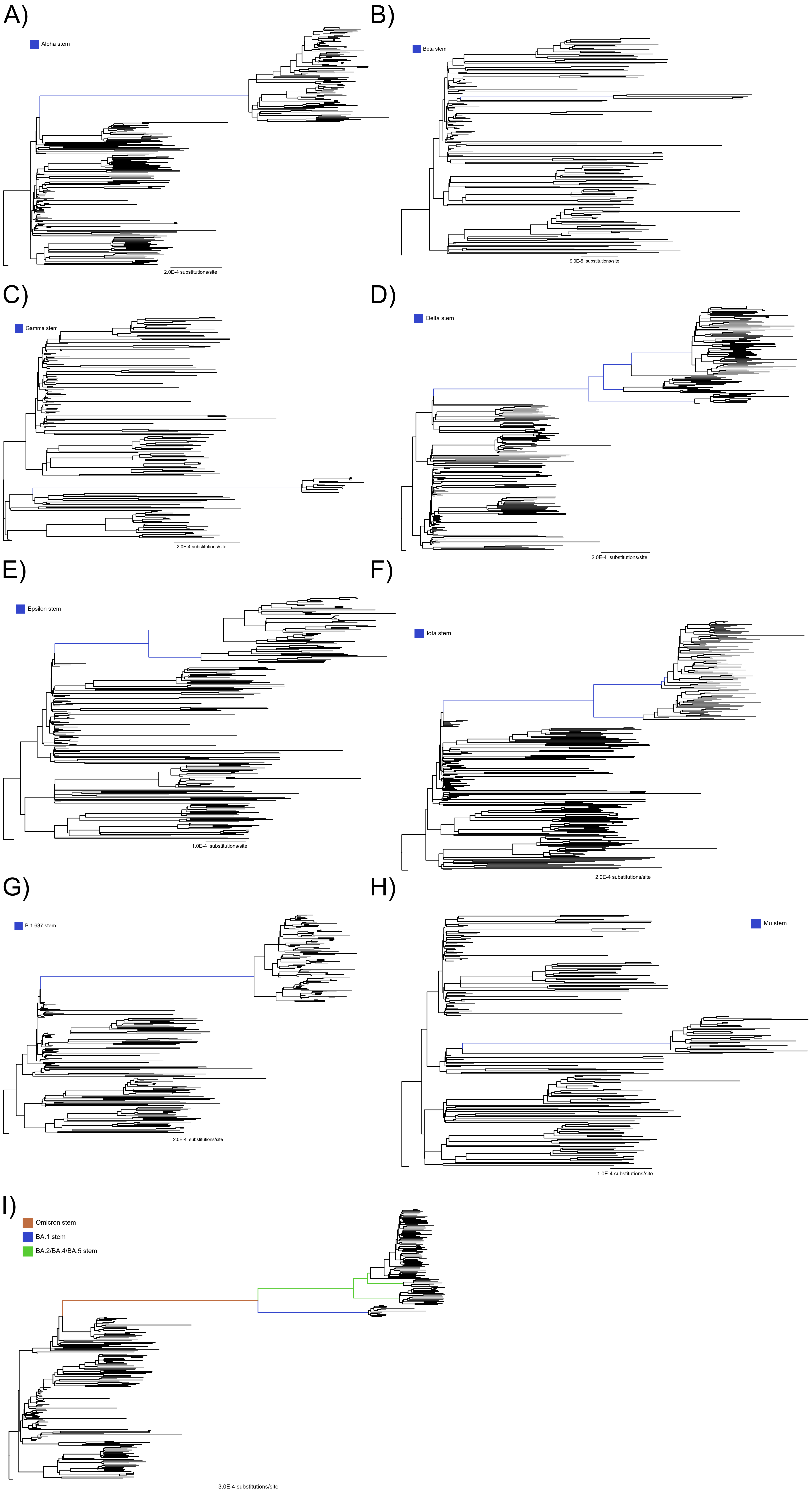
A)
Alpha stem
2.0E-4 substitutions/site
B)
Beta stem
9.0E-5 substitutions/site
C)
Gamma stem
2.0E-4 substitutions/site
D)
Delta stem
2.0E-4 substitutions/site
E)
Epsilon stem
1.0E-4 substitutions/site
F)
Iota stem
2.0E-4 substitutions/site
G)
B.1.637 stem
2.0E-4 substitutions/site
H)
Mu stem
1.0E-4 substitutions/site
I)
Omicron stem
BA.1 stem
BA.2/BA.4/BA.5 stem
3.0E-4 substitutions/site
