# Supplementary material for: Slowdown of synonymous substitution rate preceding the emergence of multiple SARS-CoV-2 variants
Source: Virus Evol. 2026 Aug 20;12(1):veag054. doi: 10.1093/ve/veag054 (PMC13580085; doi:10.1093/ve/veag054)
Supplement: Supplementary file 1 [file supplementary_material_veag054(1).zip › Supplementary_material_veag054/SuppFig2_PITrees.pdf]

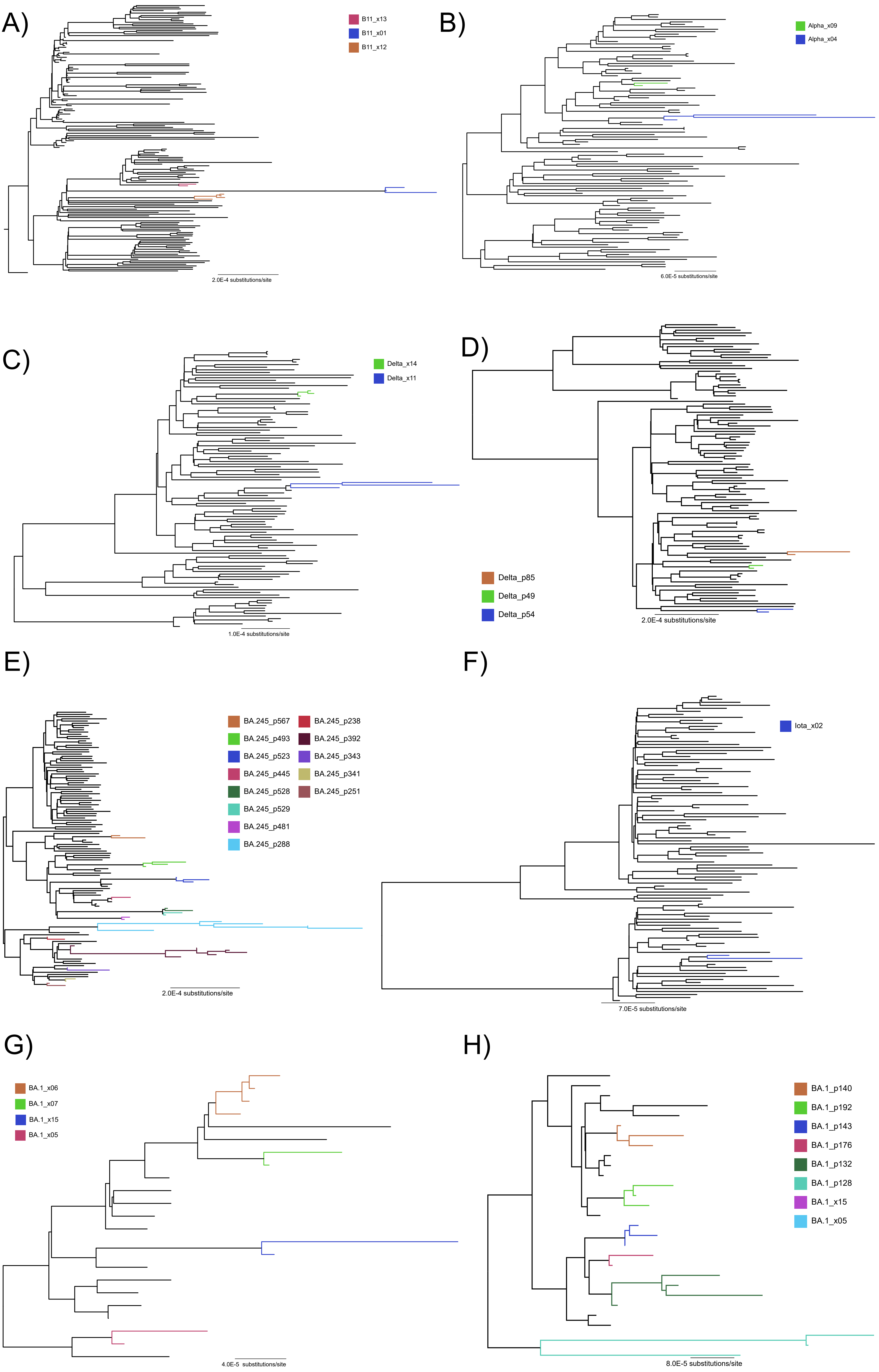

A)
B11_x13
B11_x01
B11_x12
2.0E-4 substitutions/site
B)
Alpha_x09
Alpha_x04
6.0E-5 substitutions/site
C)
Delta_x14
Delta_x11
1.0E-4 substitutions/site
D)
Delta_p85
Delta_p49
Delta_p54
2.0E-4 substitutions/site
E)
BA.245_p567
BA.245_p493
BA.245_p523
BA.245_p445
BA.245_p528
BA.245_p529
BA.245_p481
BA.245_p288
BA.245_p238
BA.245_p392
BA.245_p343
BA.245_p341
BA.245_p251
2.0E-4 substitutions/site
F)
Iota_x02
7.0E-5 substitutions/site
G)
BA.1_x06
BA.1_x07
BA.1_x15
BA.1_x05
4.0E-5 substitutions/site
H)
BA.1_p140
BA.1_p192
BA.1_p143
BA.1_p176
BA.1_p132
BA.1_p128
BA.1_x15
BA.1_x05
8.0E-5 substitutions/site
