# Supplementary material for: Slowdown of synonymous substitution rate preceding the emergence of multiple SARS-CoV-2 variants
Source: Virus Evol. 2026 Aug 20;12(1):veag054. doi: 10.1093/ve/veag054 (PMC13580085; doi:10.1093/ve/veag054)
Supplement: Supplementary file 1 [file supplementary_material_veag054(1).zip › Supplementary_material_veag054/SuppFig3_DeerMinkSuppFigTrees.pdf]

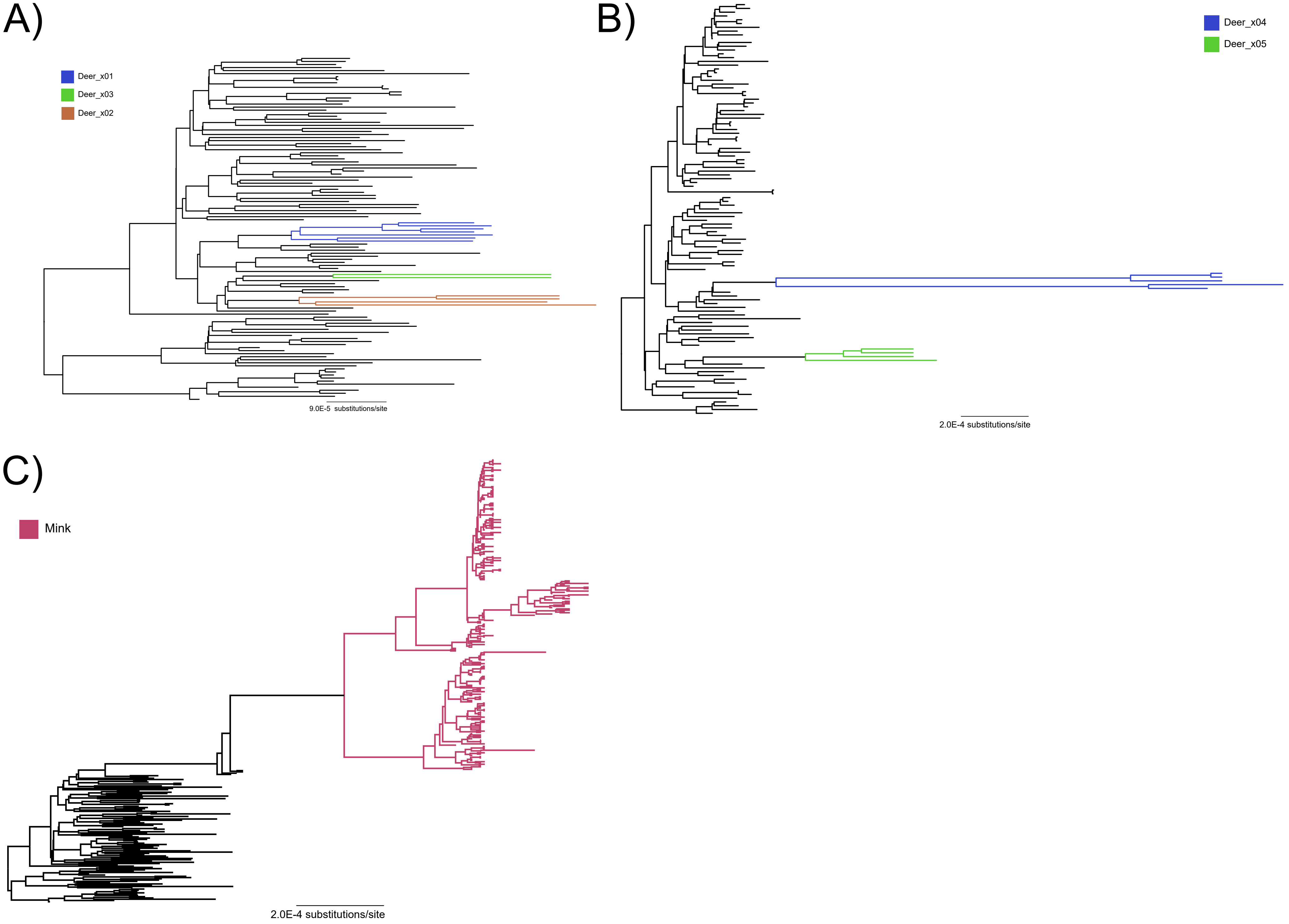

A)
Deer_x01
Deer_x03
Deer_x02
9.0E-5 substitutions/site
B)
Deer_x04
Deer_x05
2.0E-4 substitutions/site
C)
Mink
2.0E-4 substitutions/site
