# Supplementary material for: Slowdown of synonymous substitution rate preceding the emergence of multiple SARS-CoV-2 variants
Source: Virus Evol. 2026 Aug 20;12(1):veag054. doi: 10.1093/ve/veag054 (PMC13580085; doi:10.1093/ve/veag054)
Supplement: Supplementary file 1 [file supplementary_material_veag054(1).zip › Supplementary_material_veag054/SuppFig4_OverallRate.pdf]

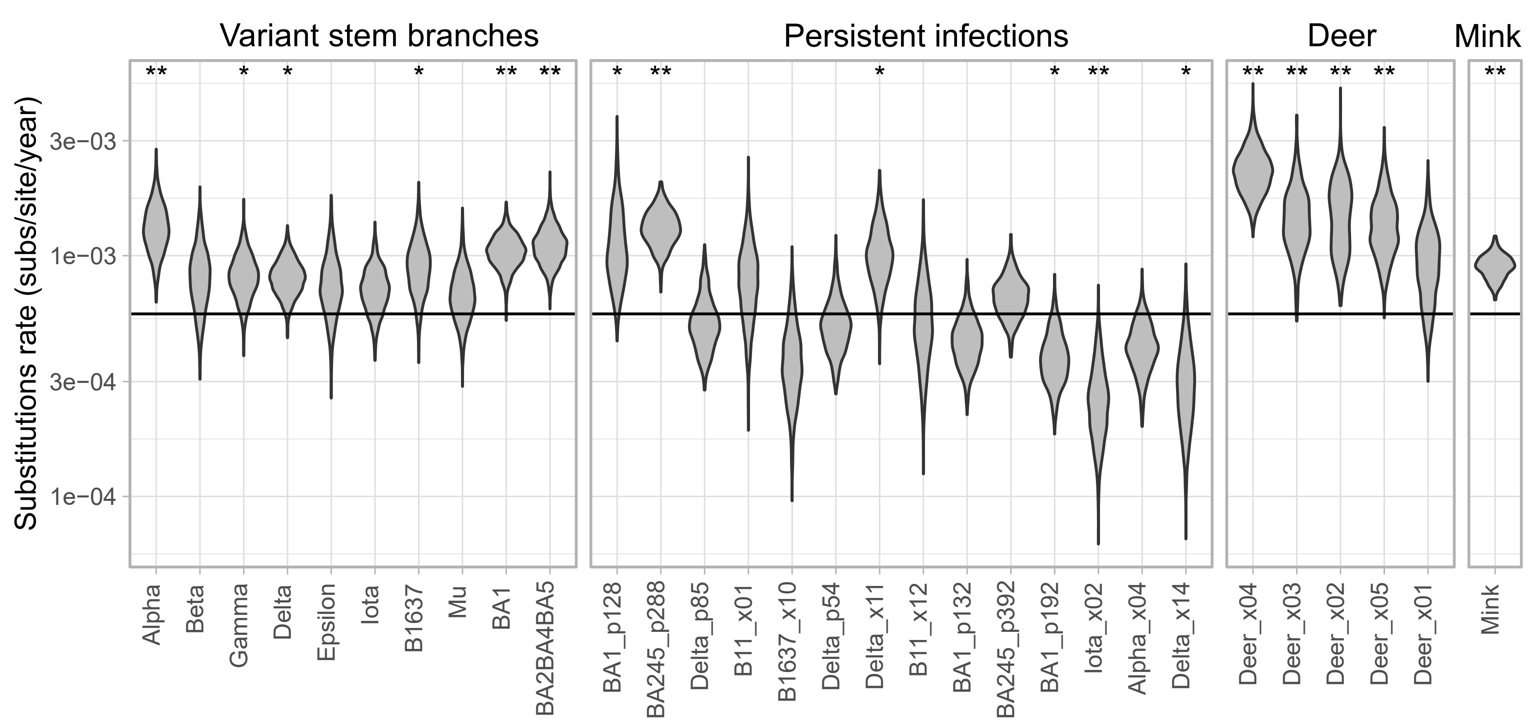

Variant stem branches
Persistent infections
Deer
Mink
Substitutions rate (subs/site/year)
3e−03
1e−03
3e−04
1e−04
** * * * ** **
* ** * * ** *
** ** ** **
**
Alpha
Beta
Gamma
Delta
Epsilon
Iota
B1637
Mu
BA1
BA2BA4BA5
BA1_p128
BA245_p288
Delta_p85
B11_x01
B1637_x10
Delta_p54
Delta_x11
B11_x12
BA1_p132
BA245_p392
BA1_p192
Iota_x02
Alpha_x04
Delta_x14
Deer_x04
Deer_x03
Deer_x02
Deer_x05
Deer_x01
Mink
