# Supplementary material for: Slowdown of synonymous substitution rate preceding the emergence of multiple SARS-CoV-2 variants
Source: Virus Evol. 2026 Aug 20;12(1):veag054. doi: 10.1093/ve/veag054 (PMC13580085; doi:10.1093/ve/veag054)
Supplement: Supplementary file 1 [file supplementary_material_veag054(1).zip › Supplementary_material_veag054/SuppFig5_p392.pdf]

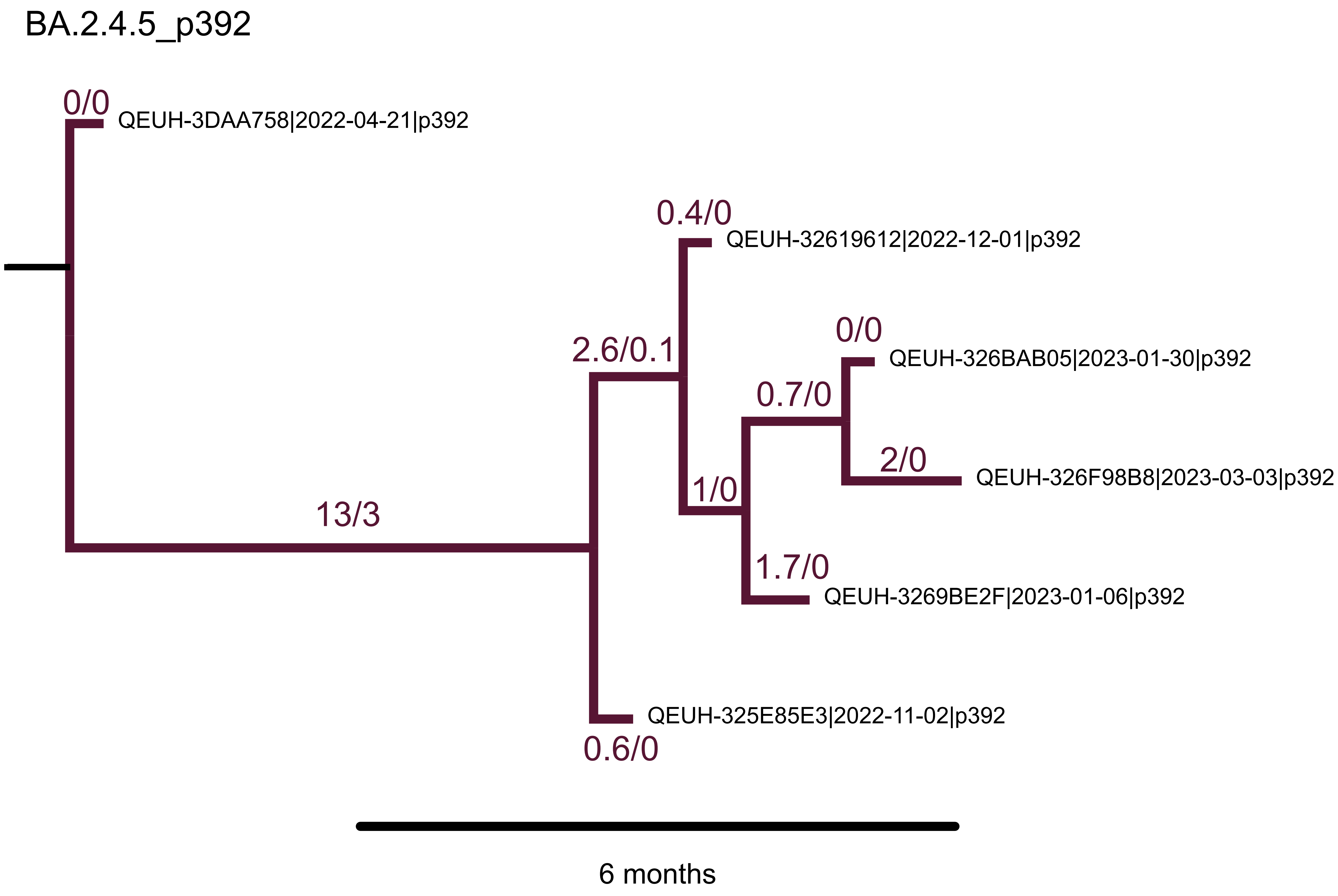
BA.2.4.5_p392
0/0
QEUH-3DAA758|2022-04-21|p392
0.4/0
QEUH-32619612|2022-12-01|p392
0/0
QEUH-326BAB05|2023-01-30|p392
2.6/0.1
0.7/0
2/0
QEUH-326F98B8|2023-03-03|p392
1/0
13/3
1.7/0
QEUH-3269BE2F|2023-01-06|p392
QEUH-325E85E3|2022-11-02|p392
0.6/0
6 months
