# Supplementary material for: Slowdown of synonymous substitution rate preceding the emergence of multiple SARS-CoV-2 variants
Source: Virus Evol. 2026 Aug 20;12(1):veag054. doi: 10.1093/ve/veag054 (PMC13580085; doi:10.1093/ve/veag054)
Supplement: Supplementary file 1 [file supplementary_material_veag054(1).zip › Supplementary_material_veag054/Supplementary information.docx]

# Supplementary information

**Supp Fig 1. Phylogenetic trees of B.1-like and VOC sequences.** The variant stem branches are highlighted in blue for A) Alpha B) Beta C) Gamma D) Delta E) Epsilon F) Iota G) B.1.637 and H) Mu. I) The shared Omicron variant stem branch is highlighted in orange, BA.2/4/5 is highlighted in green and BA.1 is highlighted in blue. The black branches represent circulating genomic diversity of B.1-like virus and variant virus.

**Supp Fig 2. Phylogenetic trees of persistent infections in background context.** Background context branches are colored in black for A) B.1-like B) Alpha C-D) Delta E) BA.2/BA.4/BA.5 F) Iota and G-H) BA.1 sequences. Individual persistent infection clades from A-C,F-G) NYC and D-E H) UK are colored.

**Supp Fig 3. Phylogenetic trees of non-human hosts and context sequences.** Human background context branches are colored in black for A,C) Delta and B) Alpha with A,B) deer and C) mink clades colored.

**Supp Fig 4. Rate of molecular clock.** A) Summary of overall molecular clock (grey) with posterior probability that the rate is larger or smaller than the background rate (* P >0.9; ** P >0.99). Background substitution rate median (5.4x10^-4^ substitutions/site/year) indicated by black horizontal line.

**Supp Fig 5. Phylogenetic trees of persistent infection BA.2.4.5_p392** with time scaled branches annotated with robust counts of nonsynonymous/synonymous substitutions.

## Supp Table 1. Molecular clock and rate of nonsynonymous and synonymous substitutions

## Supp Table 2: Synonymous substitutions of variant stem branches

## Supp Table 3. Ascension numbers and ID of context sequences

## Supp Table 4. Ascension numbers and ID of persistent infection sequences

## Supp Table 5. Ascension numbers and ID of sequences from non-human hosts

Supp Data 1-23 XMLs used for Bayesian local clock inference and robust counting
